# Cases from the Note Book

**Published:** 1855-04

**Authors:** Owen Long

**Affiliations:** Jacksonville, Ill.


ART. IV.—*Cases from the Note Book.* By OWEN LONG, M.D., of Jacksonville, Ill.

IN presenting the following cases to the readers of your Journal I would state that it is not done because there is anything new or important in them, but because it is the duty of every professional man to give his experience in the use of any new article that may be introduced to the notice of the profession.

It is several years since Anæsthetics were introduced as articles of the Materia Medica. Each year has only added to the value of that contribution, and the discoverer or discoverers should not be compelled to wait any longer for that reward which they have so richly merited. I would further state that both ether and chloroform have been freely used by physicians in this section of the State without one bad case resulting from their use; on the contrary, our experience has satisfied us that the discovery of the means of producing uncontrollable anæsthesia, has been one of the greatest blessings bestowed upon man.

Jan. 24, 1855.—Called to see Charles, a German, aged 25. He was out in the storm of Sunday, 21st January, traveling about all day. His boots being full of water, his feet soon became so much frozen that he could no longer walk in this condition. At three o'clock, P M,, he crawled into a haystack, and remained there till next morning. His hands, ears, face and feet being frozen badly, when he was brought to the Morgan county almshouse, of which I am physician. I was satisfied from inspection, that in consequence of the wet condition that his feet were in, that all vitality had been destroyed in them by the intense cold of that awfully stormy day and night. All the usual means in such cases were applied, and although I had the pleasure of seeing his face, hands, ears, etc., restored to their former condition, yet I was much pained to find, on the morning of the 4th of February, two weeks after the exposure, unmistakeable signs of gangrene; by the next day the line of demarcation was so plain and certain that upon consultation with Dr. Prince, I determined to remove both limbs as the only hope of saving my patient, who had begun most unmistakeably to exhibit symptoms of debility. The whole of both feet below the malleoli now presented the usual gangrenous appearance and from his entrance into the Institution till now, had never made any other complaint except of a very deep seatied and *heavy* pain, to use his own expression, in the middle of his foot. This I have no doubt was produced by that extreme degree of congestion, which immediately precedes gangrene, and which was confined to the inner structure of the feet.

All things being prepared he was very soon reduced to complete insensibility by the use of chloroform, having first attempted to induce anesthesia by the use of Ether, which completely failed in this case, to even partially effect him. Both legs were then amputated about four inches below the Patella, the arteries were taken up, the flaps were approximated by stitches and adhesive straps, the bandages, etc., were all applied, whilst he was still under the influence of chloroform. He was much rejoiced to find that all was over and he had not felt any pain. Is not such an article a great boon to suffering humanity?

Feb. 10, 6 P.M., 1855.—Call to see Ella, aged 30 months. She was taken with convulsions, which had continued without interruption to this time from 1 P.M. During the afternoon she had been under the care of Dr. Cassell, a very excellent and judicious practitioner, who had administered the usual means without producing any relief. Her pupils were very much dilated. As she had been freely *puked* and *purged*, I opened the temporal artery and obtained as much blood as I desired without benefit. I now learned that she had fallen from a chair upon the back of her head, receiving a very violent concussion of the brain. I was now satisfied, from the great dilatation of the pupils, that effusion had taken place, and that she was beyond the reach of all remedies. I however, with Dr. C.'s approbation, immediately put her under the influence of chloroform. The convulsions immediately ceased, and she would remain as tranquil as if in the sweetest sleep until its influence would pass off, when twitchings of the eye would indicate its return. A few inhalations however would soon tranquilize the system, and thus the hearts of her parents were saved from witnessing the cruel writhings and contortions of this excruciating disease which had afflicted the patient all afternoon. Relief from spasm of any kind is a great boon; here it is doubly so. Although we may not cure, still we may smooth the passage to the grave by the use of chloroform. In cholera nothing is more efficacious than chloroform to relieve its frightful spasms.

Feb. 12, 1855.—Called to see Mrs. C., aged 20 She had been delivered of twins, 48 hours intervening between the births. The last child had been born about thirty hours, both after-births had been retained by an hour glass contraction of the womb.

The physician in attendance failed in all his efforts to extract. External and internal organs of generation very much swollen and extremely tender to the touch, so much so that she could not allow me to introduce even my finger into the vagina. This was a very interesting case, one that would show the invaluable character of chloroform during my efforts to relieve this lady, who ever since the birth of her last child had been tormented with constant and continued efforts of the uterus to rid itself of its contents.

This patient was soon brought under its influence, the hand was readily and without difficulty or pain introduced and passed through the stricture or contraction, up to the fundus uteri, to which I now found the placenta were both firmly adhered. They were however readily removed by the operation of *peeling*, and my patient was thus rescued from the jaws of death.

I would add my advice to my professional brethren always to use ether or chloroform in all operations where much pain and suffering may be expected.

Reasoning a priori, would not anæsthetics be productive of much relief in all cases of jactitation and restlessness in fever, would not its use immediately allay all excitement, of whatever kind it might be, unless in that of a highly sthenic character, such as generally attends our fevers of an active inflammatory condition. I do not remember of having ever seen the use of chloroform advised as above, and if it has never been used I would beg leave to call the attention of the profession to this subject.

With these few items of a practical character, I leave the subject with your readers, only adding that the mode of administration has always been by wetting a handkerchief with a drachm or two of ether or chloroform, and thus applying it to the nose until its effects are produced. My rule always is to use ether, if it will answer; if it does not, then use chloroform without much delay.

---

[Dr. Davis,—Dear Sir: I think you will find the enclosed note of sufficient interest to deserve insertion in your Journal. As illustrating the existence of an animal poison hitherto unknown, or the action of an old one in an unusual manner, the case it describes deserves to be put on record, deficient as it is in details. Your obedient servant, D. Brainard.]

Dr. Brainard.—*Dear Sir:* I write to you for the purpose of obtaining some information in regard to a very singular case of supposed poisoning now in this city, under the medical care of my preceptor, J. H. Hershey and his partner, Dr. Cass. The history of the case is as follows: The patient is a b y ten years of age. He says that he was bitten by a rat in the night, while in bed There are two small wounds or marks on his upper lip, which are about the size I should suppose would be made by the teeth of the rat. The pati nt did not experience any bad effects for a week after he had been bitten, at which time the part bitte began to swell, and the swelling extended itself to the greater part of the face and neck. The part is rigid to the touch and rather insensible. All the glands of the system appear to be affected; they are swollen, causing a considerable stiffness in most of the joints; the circulation is excited, but not extremely so; the pulse rather frequent and full. It has been treated locally by the application of the tinct. iodine; constitutionally, by iodide potassa and tonics.

Now, I r., inasmuch as the attendant physicians are anxious that their patient may recover, and I am solicitous to know your opinion of the case, you will have all our thanks for any information you may be pleased to send us. In short, Dr., we want to know whether the bite of the RAT is poisonous or not.

Yours respectfully, J. J. MORGAN.

